# Posttranslational Modification of Waxy to Genetically Improve Starch Quality in Rice Grain

**DOI:** 10.3390/ijms22094845

**Published:** 2021-05-03

**Authors:** Tosin Victor Adegoke, Yifeng Wang, Lijuan Chen, Huimei Wang, Wanning Liu, Xingyong Liu, Yi-Chen Cheng, Xiaohong Tong, Jiezheng Ying, Jian Zhang

**Affiliations:** 1 State Key Lab of Rice Biology, China National Rice Research Institute, Hangzhou 311400, China; 2019Y90100074@caas.cn (T.V.A.); wangyifeng@caas.cn (Y.W.); chenlijuan0723@gmail.com (L.C.); wanghuimei@caas.cn (H.W.); liuwn123456789@gmail.com (W.L.); liuxinyong1234@gmail.com (X.L.); chengyichen0721@gmail.com (Y.-C.C.); tongxiaohong@caas.cn (X.T.); yingjiezheng@caas.cn (J.Y.); 2 Graduate School of Chinese Academy of Agricultural Sciences, Beijing 100081, China

**Keywords:** waxy, amylose, posttranslational modification, GBSSI, rice

## Abstract

The *waxy* (*Wx*) gene, encoding the granule-bound starch synthase (GBSS), is responsible for amylose biosynthesis and plays a crucial role in defining eating and cooking quality. The waxy locus controls both the non-waxy and waxy rice phenotypes. Rice starch can be altered into various forms by either reducing or increasing the amylose content, depending on consumer preference and region. Low-amylose rice is preferred by consumers because of its softness and sticky appearance. A better way of improving crops other than downregulation and overexpression of a gene or genes may be achieved through the posttranslational modification of sites or regulatory enzymes that regulate them because of their significance. The impact of posttranslational GBSSI modifications on extra-long unit chains (ELCs) remains largely unknown. Numerous studies have been reported on different crops, such as wheat, maize, and barley, but the rice starch granule proteome remains largely unknown. There is a need to improve the yield of low-amylose rice by employing posttranslational modification of *Wx*, since the market demand is increasing every day in order to meet the market demand for low-amylose rice in the regional area that prefers low-amylose rice, particularly in China. In this review, we have conducted an in-depth review of waxy rice, starch properties, starch biosynthesis, and posttranslational modification of waxy protein to genetically improve starch quality in rice grains.

## 1. Introduction

Rice (*Oryza sativa* L.), one of the most vital crops, is a primary meal for more than half of the world’s population and also serves as a source of energy and nutrition for millions of consumers. It is a significant staple food in Asia, West Africa, Latin America, and the Caribbean; the main end-use of rice is human consumption [1]. By 2027, it is expected that total rice consumption will increase by 13% [1]. Its function in food processing is significant, particularly in Asia, including China [2]. About 70% of the expected rise in global rice demand is accounted for by Asian nations, primarily due to growth in population rather than per capita demand [1]. The rice per capita consumption in kg/person/year in 2014–2016 was 77.8 and will increase to 78.9 in 2026 at 0.08% growth increase per annum in Asia and Pacific [1]. As China’s population expands, by 2030, China would have to generate 20% more rice to satisfy its domestic needs if the rice per capita demand remains at the current pace [3]. According to Guo et al. [4], China’s population will increase to 1.458 billion in 2030 from 1.33 billion in 2010 if moderate growth is maintained. By 2035, it will increase to about 1.46 billion and then decline to 1.38 billion by 2050 [4]. Since China is a regional area that prefers waxy and low-amylose rice, more energy should be channeled on how to improve the yield of such varieties.

**Figure 1:**
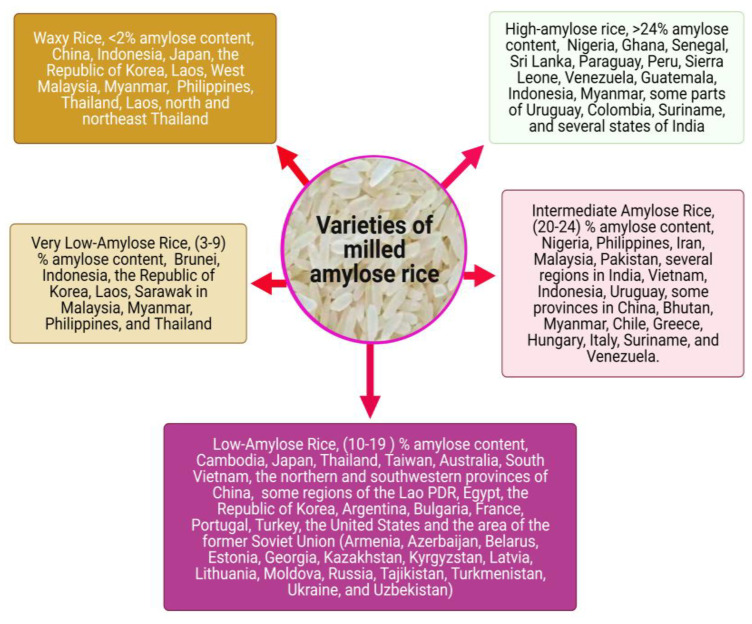
Varieties of amylose rice regionally preferred by different countries. Classification of milled rice based on amylose content and the list of countries that preferred different varieties of amylose rice (created with BioRender.com).

The primary carbohydrate in rice is starch, which ranges from 72 to 75% [5], while the protein content of 3805 *Indica* varieties in China ranged from 6.3 to 15.7%, and that of 1518 *Japonica* varieties ranged from 6.0 to 13.6% [6]. The rice quality is mainly determined by the starch content, especially the cooking and eating qualities [7]. Sun et al. [8] also reported that starch properties primarily affect rice-eating quality. Starch is an essential resource for humans and industries and is abundantly present in many varieties of starch-storing crops, e.g., tubers, storage roots, and cereal seeds [9]. Regina et al. [10] also reported that it is commercially isolated from a wide variety of crops, including stem and pith (e.g., sago), roots and tubers (e.g., cassava, sweet potatoes, potatoes, and arrows), and whole grains (e.g., rice, wheat, and sorghum). Furthermore, starch is a cheap, biodegradable, and sustainable industrial raw material that provides sufficient calories for humans and animals [9]. Consumers are changing their eating habits to incorporate rice varieties with good cooking and eating qualities [11]. Cooking and eating properties are closely related to water absorption, increase in volume, and overall firmness of cooked rice. For eating quality and market approval, rice texture is of vital importance [12]; it is a sensory property that influences the stiffness, stickiness, and overall texture of cooked rice [13]. Chen et al. [14] observed that in addition to eating quality and consumer preference, industries have also employed grain starch as an adhesion, sizing, gelling, thickening, and binding agent. Starch obtained from rice has been used in various foodstuffs and consumer items, such as dessert, baking products, and fats, owing to a broad variety of amylose levels [15]. Champagne reported that “the amylose content of milled rice varies from 0.8 to 37% among varieties” [16]. However, the classification of Juliano varies from less than 2% to 33% and consists of the waxy, very low, low, intermediate, and high amylose classes of rice with amylose contents of <2%, 2–12%, 12–20%, 20–25%, and 25–33%, respectively [17]. The amylose content of milled rice can be described as follows: high, >25.0%; intermediate, 20.1–25.0%; low, 12.1–20.0%; very low, 5.1–12.0%; and waxy, 0–5% [18]. Chen et al. [19] also reported that milled rice amylose is typically classified into five classes: (i) high amylose content, (ii) intermediate, (iii) low, (iv) very low, and (v) waxy, with amylose contents of ˃24%, 20–24%, 10–19%, 3–9%, and 0–2%, respectively. Consumers in China, Indonesia, the Philippines, Lao PDR, Myanmar, the Republic of Korea, Thailand, Japan, and West Malaysia were found to choose sticky or waxy rice over other classes of waxy rice [18], and this result was confirmed by another study among consumers in Lao PDR and Isan, Thailand [11]. In Indonesia, Myanmar, the Philippines, Lao PDR, the Republic of Korea, Brunei, Thailand, and Sarawak in Malaysia, very low amylose rice is preferred by consumers [18]. Low-amylose rice was found to be preferred by consumers in Cambodia, Japan, Thailand, Taiwan, Australia, South Vietnam, northern and southwestern provinces of China, some regions of Lao PDR, and Egypt [11]; in a separate study, the preference for low-amylose rice was recorded for consumers in Taiwan, Turkey, the Republic of Korea, Cambodia, Egypt, France, Japan, Thailand, Portugal, Australia, South Vietnam, some regions of Lao PDR, Argentina, Bulgaria, the northern and southwestern provinces of China, the United States, and the former Soviet Union (Moldova, Georgia, Uzbekistan, Lithuania, Ukraine, Russia, Azerbaijan, Kyrgyzstan, Turkmenistan, Armenia, Estonia, Belarus, Kazakhstan, Tajikistan, and Latvia) [18]. In the Philippines, Iran, Malaysia, Pakistan, several regions in India, Vietnam, Indonesia, Uruguay, and some provinces in China, intermediate amylose rice is preferable [11]. Juliano [18] reported that intermediate amylose is preferable in Nigeria, Hungary, Bhutan, Philippines, Uruguay, Iran, Italy, Greece, Suriname, several regions in India, Vietnam, Pakistan, Indonesia, Malaysia, some provinces in China, Myanmar, Chile, and Venezuela. High-amylose rice is regionally preferred in Senegal, Sri Lanka, Indonesia, Myanmar, and some parts of Uruguay, Colombia, Ghana, and Suriname, in several states of India [11]. In Nigeria, Paraguay, Peru, in several states of India, Ghana, Guatemala, Colombia, Senegal, some parts of Uruguay, Sierra Leone, Venezuela, Suriname, Indonesia, Myanmar, and Sri Lanka, high-amylose rice is desirable/selected [18]. Few countries preferred more than one type of milled rice classification based on the amylose content. Varieties of amylose rice are regionally preferred by different countries (Figure 1).

The textural characteristics of cooked rice are mostly distinguished by the content of the starch material, because starch is an essential part of the rice endosperm [20]. The proportions of the short to long chains of waxy rice grains were slightly smaller than those of other grain crop classes [20,21]. The amylose content in the grains is considered an essential factor in the characteristics of cultivated rice. Most efforts to elevate or boost the composition of starch are to increase the ADP-glucose (ADPG) amount required for the biosynthesis of starch [10]. Earlier work included the expression of ADP-glucose pyrophosphorylase-mutated bacteria with decreased allosteric dependency on fructose-6-phosphate activator in potatoes, leading to a rise of >35% in potato tuber starch relative to wild type [22].

Waxy rice is the primary focus of plant breeders in China and is generally used in brewing and conventional Chinese cuisine [23]. The development of amylose is the main determining factor of cooking and eating quality, and the waxy (*Wx*) protein in rice grains is crucial for amylose synthesis [24]. For both gel consistency and amylose content, *waxy* (GBSSI) serves as a vital/critical gene, but it has a minor influence on the temperature of gelatinization [8]. The absence of amylose contributes to comparatively simple starch gelatinization and a lower retrograde tendency, and both results raise the sensitivity of starch to hydrolyzing enzymes with increased digestibility [25]. Granule-bound starch synthase (GBSS) is widely acknowledged to be responsible for amylose synthesis [23]. The GBSS of cereals is subdivided into GBSSI and GBSSII [26]. GBSSI controls the synthesis of amylose in storage tissues (e.g., seed endosperm) [26]. In storage tissues (e.g., seed endosperm), GBSSI is responsible for amylose synthesis [23,27], while GBSSII exists in the green tissues, including the seed pericarp [23,27]. Waxy loci encode granule-bound starch synthase I (GBSSI), which controls the elongation of the endosperm kernel’s amylose [25]. Downregulation of GBSSI reduces amylose content and improves the quality of cooking and eating properties [28]. The non-existence or inactivation of waxy (*Wx*) locus-coded GBSSI in plants gives rise to low waxy or waxy rice [29]. Overexpression and downregulation of single or multiple genes have been predominantly crop improvement approaches employed over the past decades [30]. Although some significant advancements have been made, these are comparatively brute force methods, which sometimes contribute to unwanted trade-offs between plant development and stress resilience [30].

In addition to the approaches listed above, a better way of improving crops may be achieved through the posttranslational modification (PTM) of sites or regulatory enzymes that regulate them because of their significance [30] and also because proteins are essential for cell phenotype [31]. Komatsu et al. [32] reported that the ability to discover posttranslational modification (PTM) sites, which is needed to assess the functional effect of protein modification on crop productivity, is a distinct benefit of proteomics over other “omics” techniques. However, genomic editing approaches and transgenic technologies have led to the discovery of novel phenotypes [33]. These methods add new alleles that evoke and enhance stimuli response, resulting in a phenotype improvement; however, the entirety of gene response pathways is still unknown [33]. New strategies must be explored to increase the capacity for plant phenotype improvement [33]. While genomic research can help scientists understand what is potentially conceivable, proteomic research reveals the practical players involved in complex cellular processes [34]. Although functional genomics is effective in elucidating the function of genes, it is necessary to use them in relation to producing a specific phenotype [33]. PTM of proteins is one such avenue. By triggering or repressing protein expression, this mechanism adds another layer of regulation to gene response and allows for fine-tuning of gene response pathways [33]. Graves and Haystead [31] reported that proteomics techniques include PTMs, protein–protein interactions, structural proteomics, functional proteomics, protein mining, and protein expression profiling. Two-dimensional polyacrylamide gel electrophoresis (2D-PAGE) and gel-free-based shotgun procedures have been used extensively in the study of rice grain proteomics [35,36]. Two-dimensional electrophoresis (2-DE) gel is the most commonly employed method for distinguishing proteins in the majority of cited articles [32]. However, in several laboratories, liquid chromatography (LC)-based proteomic analysis is becoming more popular [32]. Both protein separation strategies have their own set of benefits [32]. Protein alteration and degradation can be quickly visualized using a conventional 2-DE technique, while LC-based approaches need a small amount of starting material [32]. Proteomics approaches are great molecular tools that have been extensively employed to explore the molecular basis of many biological mechanisms in plants [37]. The starch granular proteomic study is essential for understanding the starch biosynthesis pathway and its packaging in the amyloplasts of rice in order to improve the quality of grains [38]. Helle et al. [39] also reported that starch granular growth and structural design may be affected by the starch proteome, which leads to a better understanding of starch biosynthesis. Quantitative proteomics has been employed for protein identification and validation of trait-specific markers by coordinating changes in protein levels [40]. To generate mature proteoforms that gradually accumulate in plant cells to form the observed proteome, several proteins undergo PTMs [41,42]. Numerous PTMs have been associated with a wide variety of metabolic roles in recent large-scale proteomic experiments [43].

Posttranslational modification (PTM) processes play vital roles in determining the functional performance of the genome and gene transcription [30]. Virtually any part of protein behavior may be controlled by PTM, including subcellular localization, networks of protein–protein interactions, enzymatic activity, and protein stability [44]. The impact of posttranslational GBSSI modifications on extra-long unit chains (ELCs) remains undetermined, and since serine and tyrosine can be phosphorylated, the importance of posttranslational modifications becomes more noticeable [45]. PTMs have been employed to alter starch quality in various crops to produce different varieties of amylose grains and tubers, depending on consumer preferences and needs. Numerous studies have been reported on various crops, such as wheat, maize, and barley, but the rice starch granule proteome remains largely unknown. However, a large-scale phosphoproteomic study of starch granule binding proteins in cereal crops has not been conducted because of two main setbacks [46]: (i) due to the very low protein content and the high sugar content of the granules and the existence of various compounds that interfere with protein extraction, it is challenging to enrich sufficient proteins from the starch granules for phosphoproteomic analysis, and (ii) there is limited knowledge of phosphorylation modifications in various crops, especially in the very large hexaploid wheat genome, which is ~17 Gb.

In this review, we have conducted an in-depth review of waxy rice, starch properties, starch biosynthesis, and posttranslational modification of waxy protein to genetically improve starch quality in rice grains.

## 2. Starch Properties

Starch, the primary carbohydrate content in plants, is a crucial natural source of feed, raw materials for industry, and food [9]. It plays a crucial role as the main source of stored carbohydrates for chemical energy in the life cycle of the leaf [47]. To fulfill the continuing energy needs of plant growth (e.g., during the diurnal leaf cycle), transient starch is generated and deteriorates quickly [47]. Conversely, in expectation of potential plant energy demands, for example, germination of seeds or sprouting of the tuber, storage starch accumulates and survives in heterotrophic tissues [47]. It is conserved in sink tissues as an energy source [48]. It exists in the photosynthetic and non-photosynthetic tissue plastids [49]. Starch, the key reserved energy and carbohydrate in the plant, may be classified into two forms, storage starch and transient starch, in relation to their biological role [9]. During the day, transient starch is stored in chloroplasts in photosynthetic tissues [9], and during the night, growth and metabolism are due to transient starch being transferred and depleted to provide nutritious materials and energy [9]. In non-photosynthetic tissues, specialized plastids known as amyloplasts (e.g., storage roots, tubers, and endosperm seeds) store starches that are reserved for a long time [9], and in readiness for propagation, regrowth, or sprouting, they can be remobilized [9].

Starch has two major constituents: α-polyglucan amylose, which is essentially linear, and α-polyglucan amylopectin, which is branched [50,51]. The natural starch composition is uniform with an amylopectin component of 75% and a minor component of amylose of 25%, regardless of the source [10]. The polymers of α-1,4-linked glucan chains of various proportions of α-1,6-linked branch points are both amylose and amylopectin [10]. Although amylose is largely linear with a linkage of about 1% α-1,6, amylopectin is a much bigger and strongly branched molecule with 4–5% α-1,6 connections [10]. Amylopectin has a far more established structure, called the tandem cluster, than glycogen because it consists of tandem-linked clusters (approximately 9–10 nm in length each), where linear α-1,4-glucan chains are regularly branched through α-1,6-glucosidic linkages, as compared to bacterial and animal glycogens which have a more randomly branched structure [51,52].

Starch modification includes an effort to boost or minimize the starch content and adjust the composition and component structure to improve the starch properties by reducing or increasing its content to satisfy consumer preferences and suit particular end uses in industries [10]. Three target points were previously taken to increase starch production in plants: downstream starch biosynthetic enzymes, rate-limiting steps in AGPase-containing starch biosynthesis, and precursor molecules for starch biosynthesis [10]. The physicochemical properties of gelatinized starch are defined primarily by starch properties, and the composition of lipid, protein, and starch granule could also alter the rheological characteristics of cooked starch by reacting with amylose and probably amylopectin [53,54]. The physicochemical characteristics of starch are strongly dependent on its structural characteristics, which affect its activity during production [10]. There has been significant development throughout the last few decades to alter starch amylose content in an essential cereal, either by reducing or increasing the AC compared to the wild-type amylose content [10]. The amylose–amylopectin ratio is the most widely discussed target for starch modification [55]. It is well known that the significant factor affecting the starch physicochemical properties is the amylose-amylopectin ratio [20]. Amylose is capable of forming a solid gel, whereas amylopectin exhibits little gel contraction and great retrograde resistance [20]. GBSSI, starch branching enzymes (SBEs), and soluble starch synthases (SSSs) affect starch synthesis in cereals [56]; GBSSI is involved in amylose synthesis [56,57], while SBE, SS, and DBE affect amylopectin synthesis [58]. Hanashiro et al. [59] reported that GBSSI is responsible not only for the synthesis of amylose but also for the synthesis of amylopectin, particularly for the development of extra-long-chain amylopectin. There is minimal understanding of the starch biosynthesis pathway of biosynthetic starch and the various enzymes involved in this process [9]. Several plants have been improved to develop both high-amylose and high-amylopectin starches via biosynthetic pathways [10]. In raw rice, a higher amylose content is responsible for less sticky and firm rice after cooking [60]. According to recent research, stickiness and amylose content are often negatively associated [61]. Amylose is a straight and long starch molecule that does not gelatinize during cooking. The grain final yield, weight, and grain quality were determined by rice grain filling [56]. Amylopectin is a starch molecule that is strongly branched and is responsible for making rice gelatinous and sticky. When cooked, high-amylopectin rice becomes very sticky, producing high starch content. Typically, short-grain rice has the lowest amylose and amylopectin content (e.g., short grain, Asian-style rice). Stickiness has been shown to improve with a decrease in the amylose content of the whole grain and with a boost in the total volume of amylopectin in leachate, the proportion of small chains of amylopectin, and amylopectin molecular size [12]. Recent research has revealed that amylose content and stiffness are often adversely associated with stickiness; that is, high-amylose rice is firmer and has low stickiness properties, while rice with low amylose content is stickier and softer [13]. The amylopectin-rich *Indica* varieties (waxy) are more resistant to rapid amylase hydrolysis and therefore have a high glycemic index (GI). In the management of diabetes and other diet- or lifestyle-related diseases, in which there is a focus on slowing down digestion of starch and delaying the pace at which glucose breaks down, significant attention has been paid to resistant starch (RS).

## 3. Waxy Protein

The amylose quality of starch is influenced by many mutated rice genes [20], but the most important gene is the *waxy* gene. Huang et al. [62] reported that *waxy* gene have been prolonged used for the modification of amylose content, and most plant breeders commonly targeted this gene for starch improvement in plants. The starch of waxy mutant contains amylopectin and amylose [20]. Low-amylose rice cultivars displayed higher peak viscosity, and the firmness of the rice flour paste was negatively correlated with the maximum viscosity of the rice starch paste, while the firmness of the paste of the rice flour and the paste of the starch correlated positively with the minimum viscosity of the rice starch paste [21]. Amylose content in rice also defines the transparent properties of the seed; for example, rice with an amylose content of more than 12% is transparent, rice with an amylose content of 8–12% is semi-translucent, and low-amylose (amylose content of less than 8%) is dull or opaque color [62]. The *Wx* gene encoding the enzyme GBSSI primarily regulates the synthesis of amylose in seed production, and the amount of amylose in the grain is closely correlated with the amount of GBSSI in the endosperm seed [63]. The waxy gene is positioned on chromosome 6 and consists of 12 introns and 13 exons [64]. There are two functional alleles in the rice waxy locus, namely (a) *Wx*^a^ and (b) *Wx*^b^, which is distinct from a significant discrepancy in the gene quality responsible for *Wx* content in mature seeds [65]. Rice *Wx* protein control is characterized by two functional alleles identified based on *Wx* protein quantity that accumulates in mature seeds [66], including *Wx*^a^, mainly found in *Indica* rice, and *Wx*^b^, usually found in *Japonica* rice, and is found to prevail in high- and low-amylose rice at the waxy locus, respectively [67]. Zhang et al. [24] reported that numerous allelic variations of *Wx* include *Wx*^a^, *Wx*^b^, *Wx*^in^, *Wx*^op^, *Wx*^mp^, and *Wx*; these variations influenced the geographical variance in amylose content and consumer preferences depending on the region. Waxy proteins, the major regulators of amylose biosynthesis, have been documented [68,69]. Zhang et al. [24] submitted that the *Wx*^a^ allele in the grain is responsible for high amylose content, while the *Wx*^b^ allele in the grain is responsible for low to moderate amylose content. The allele that regulates the generation of minor/curtailed levels of *Wx* protein is *Wx*^b^, which is primarily expressed in *O. sativa* subsp. *Japonica* [65]. The *Wx*^a^ allele generates approximately ten times more *Wx* protein than *Wx*^b^ and is commonly dispersed in domestic rice, such as *O. indica sativa*, *O. glaberrim*, and the wild progenitors thereof [65]. *Wx*^b^ synthesizes little amylose owing to the mutation on the 5À intron 1 splice site [70]. There are two different pathways by which amylopectin and amylose can be synthesized [71]. Active GBSS is essential for amylose synthesis, while amylopectin is synthesized as a result of a complex pathway involving various isoforms of starch-debranching enzymes (SDBEs), starch branching enzymes (SBEs), and starch synthase [72]. Molecular biologists are now well aware that the absence or inactivation of the *Wx* locus encoding the GBSSI in plants leads to crops with low or no amylose in storage tissues, without apparent total starch content [10]. Park et al. [73] observed that downregulation of GBSSI utilizing a three-prime untranslated region area (3′-UTR) RNA interference (RNAi) structure resulted in a low amylose content (from 5.9 to 9.0%) in transgenic rice lines, in contrast to the wild type with an amylose content of 17.7–18.0%. From this line, starch had no amylose and incredibly short-chain amylopectin, which reduced the number of gel contractions to nearly zero even after many freeze–thaw cycles [10]. Heilersig et al. [74] tested large inverted repeats consisting of the 5’ and 3’ halves of the GBSSI cDNA in potato, and their results showed that the 3IR construct resulted in a notably lower silencing efficacy than the 5IR construct and vice versa. Most waxy and low apparent amylose content (AAC) cultivars verified so far bear this polymorphism, resulting in decreased pre-mRNA splicing efficiency and encouraging alternate splicing at exon 1 cryptic locations, leading to decreased functional enzyme output and triggering the phenotypes of glutinous and low amylose [64]. Maize is the most common crop in which the waxy protein has been modified [10]. It is the most popular crop that is modified to generate waxy corn (protein) [10]. Waxy maize or glutinous maize is a form of cultivated maize categorized by stickiness when cooked because of higher amounts of amylopectin. Waxy kernels from maize plants were first observed in China in 1909. In other maize breeding programs, American breeders used this to tag hidden genes as a genetic marker for a long time because the waxy maize showed several important traits. In 1922 a researcher noted that the endosperm of waxy corn is amylose-free but contains amylopectin only, in contrast to wild maize varieties that contain both. However, the demand for waxy starches has recently increased due to consumer preference for cooking and eating quality and for potato free of amylose starches arising in European countries. AVEBE is an industrialized variety of waxy potato Eliane utilizing conventional mutation breeding methods, while BASF bred Amflora, a genetically modified potato form, by downregulating GBSSI [10]. In rice, a 50-leader/first-intron splicing site G/T polymorphism controls the development of mature GBSS messenger ribonucleic acid (mRNA), which affects amylose content [10]. The existence of G at the splicing position promotes normal splicing, leading to GBSS enhancement and high levels of amylose in *Indica,* while the T present at the junction leads to cryptic splicing, lower GBSS performance, and low amylose levels in *Japonica* [75]. To improve amylose quality, starch is synthesized to generate amylose either by overexpression of sufficient GBSSI [70] or by downregulating the activities of enzymes responsible for the biosynthesis of amylopectin [76,77]. Suppression of SBE and SSIIa in cereals results in higher resistant starch (RS) (e.g., barley and wheat) [78]. The amylose group is correlated with polymorphisms of the waxy protein (*Wx*) [66], which encodes the GBSSI enzyme and controls the synthesis of amylose [79].

## 4. Other Genes Involved in Starch Modification

Tian et al. [69] reported that in 70 rice varieties, an interaction study of 18 genes involved in gelatinization temperature, starch synthesis, gel consistency, and amylose content revealed that *waxy* (GBSSI) and *ALK* (*SSS2A*) are the major genes responsible for determining the nature of rice cooking and eating quality by influencing the gel consistency, amylose content, and temperature of gelatinization temperature. *ALK* is a major gene that regulates the temperature of grain gelatinization and a minor gene, affects the quality of the gel and amylose content [8]. The genes that influence both gelatinization temperature and gel consistency at the same time are *ISA* and *SBE3* [8]. Two main genes, *SSIIa* and *Wx*, and a variety of minor genes, *SSIV*, *SBEI*, *isoamylase*, and *SBEII3*, influence eating and cooking quality have been identified in rice through the study of various varieties [69,80]. In addition to the major genes, the minor genes included *AGPlar*, *APGiso*, *AGPsma*, *SS1*, *S-II-2*, *SSII-1*, *SSIII-2*, *SSIII-1*, *SSIV-2*, *SSIV-1*, *SBE1*, *SBE3*, *SBE4*, *ISA*, *PUL*, and *GBSSII* [69]. The functions of genes involved in starch modification include amylose synthesis, gel consistency, gel temperature, grain palatability, and amylopectin synthesis (Table 1). Tian et al. [69] stated that on the basis of interaction sites, each of the minor genes that affect eating and cooking quality could be divided into two haplotypes, namely, haplotype I and haplotype II. Haplotypes of individual genes involved in starch synthesis pathways, determined on the basis of interaction sites in 70 rice varieties studied include AGPlar-1633, AGPiso-511, SBE3+3577, *Wx*-1160+111, ISA-1499, ISA-1326, PUL+855, SSII-3+3796, SSI+3216, SSIV-2+437, and SSIII-2-1078 [69], with each of the genes classified as either haplotype I or haplotype II. Starch synthesized from sucrose is regulated by several genes, including *SuS2*, *SuS4*, *SuS3*, *AGPL2*, *AGPL1*, *AGPS*1, *SSSII-3*, *SSSI*, *SSSIII-2*, *SBEI*, *SBEIV*, and *SBEIII* [51,81]. In addition, many minor genes have unique properties; for example, *PUL*, *SSS3A*, *SSS1*, and *AGPlar* affect the amylose level, *AGPiso* affects the quality of gelatinization, and *SSS4B* (*SSSIV-2*) affects the temperature of gelatinization [8]. Correlations between gel consistency, gelatinization temperature, and amylose content are triggered by the combined activity of these relevant genes [8]. The estimation of the haplotype I amylose content is substantially higher than that of haplotype II under the regulation of the *Wx* haplotype for each related gene and vice versa [69]. The measurement of the gelatinization consistency value of haplotype II for each additive gene was substantially higher than that of haplotype I and vice versa, whereas the *Wx* haplotype was controlled [69]. *SSII-3* has a paramount significance for the gelatinization temperature properties of GT, which suggests that *SSII-3* performs a vital role in controlling the gelatinization temperature [69]. There are two types of *SSII-3* alleles: *SSII-3-II* and *SSII-3-I* [69]. Categories with *SSII-3-II* had GT values less than those of *SSII-3-I* [69]. In addition to *SSII-3*, a further study established *ISA*, *SSIV-2*, and *Wx* as minor genes influencing gelatinization temperature additively [69]. Grain palatability decreases with the downregulation of *SBE1* and *SBE3* levels, while overexpression of *SBE1* and *SBE3* increases the palatability of cereal [8]. Collectively, overexpression of *SSS1*, *SSS2A*, *SBE1*, and *SBE3*, especially *SBE3* and *SBE1*, could improve eating quality, but overexpression of GBSSI diminished the eating quality of cereal [8], while downregulation of GBSSI improved cooking and eating quality. Despite the existence of two additional *SSS2* genes, two *SSS3* genes, one *SSS1* gene, and two *SSS4* genes in rice plants, the *SSS2A* action specifies the form of amylopectin formation of rice starch to be either the *Japonica* or *Indica* type by performing a particular function in long B1 chain synthesis by extending the short A and B1 chains [82]. Guo et al. [83] used homozygous mutants to examine the basic function of *TaSSIVb-D* in starch granule synthesis in leaves at various developmental stages and reported that *TaSSIVb-D* mutations decreased the expression of genes and the amount of leaf starch granules.

The transformation of wheat with a changed variant of maize *shrunken 2* gene encodes a modified broad AGP subunit with reduced sensitivity to its adverse allosteric effector, orthophosphate, and more robust connection with small and large subunits, leading to an increased weight of grain per plant up to 38% [84]. Overexpression of *shrunken 2* (encoding large AGPase subunits) and *brittle 2* (encoding small AGPase subunits) at the same time in maize raised the starch content to a higher degree than when both genes were expressed individually [48].

Numerous reports have shown that plant hormone control genes encode enzymes that are essential for starch synthesis enzymes [81]. The production of starch synthesis genes and their enzyme functions are adversely affected by higher levels of abscisic acid (ABA) and ethylene in lower spikelets, resulting in low grain-filling values [85]. Grain filling is an accumulation of starch in the seed endosperm, and starch contributes 80–90% of an unpolished grain’s final dry weight in rice [86]. In regulating grain filling, plant hormones, particularly ABA and ethylene, play significant roles [85]. During the seed development stage, higher levels of ethylene are negatively connected to enzyme activity linked to metabolism and contribute to poor grain filling [85,87]. Exogenous ABA supplies decrease the sucrose (SUS) mRNA levels and enzyme activity of the mRNA in different rice grains, whereas the expression of the majority of starch synthetic genes is suppressed by the exogenous supply of ABA by ethephone; these genes are *SUS*, *AGPase*, and *SSS*, and they are depleted by their enzyme activity [85]. The effect of sucrose synthase (SUS) and the manifestation of the gene involved in starch synthesis are improved by a suitable concentration of exogenous abscisic acid, thus improving rice yields [87]. The transportation of sucrose into the grain is reduced by a high concentration of ABA, which reduces the capability of grains to synthesize starch [88], while a suitable ABA concentration boosts the SUS activity [89], increases gene expression related to starch metabolism [90], and improves the yield of grains [87]. The internal starch granule associated proteins in crops[91], are listed in Table 2.

## 5. Starch Biosynthesis Pathway

Starch biosynthesis from sucrose mainly influences the yield of cereal and rice quality in the developing endosperm [85]. It is a dynamic and highly coordinated mechanism that involves synchronized activities among several enzymes, such as starch debranching enzyme (DBE), starch synthase (SS), starch branching enzyme (SBE), and ADP-glucose pyrophosphorylase (AGPase), to organize activities [9]. According to the Uniprot database (https://www.uniprot.org/keywords/KW-0750 (accessed on the 20 January 2021)), starch (glucans and amylopectins) is synthesized via the ADP-glucose pathway by three main enzymes: (1) starch synthase, (2) ADP-glucose pyrophosphorylase, and (3) starch branching enzyme. To generate amylopectins, the arbitrarily branched glucan molecules are precisely debranched through the debranching enzyme. Both glucose-derived sucrose and glucose-derived fructose are synthesized in the cytoplasm and subsequently transferred to the cytosol for sucrose degradation by the invertase enzyme [8]. Starch is synthesized by multiple isoforms or subunits of five enzyme groups utilizing degraded sucrose, UDP-glucose, and fructose products, which include (1) granule-bound starch synthase (GBSS), (2) soluble starch synthase (SSS), (3) ADP-glucose pyrophosphorylase (AGP), (4) starch debranching enzyme (DBE), and (5) starch branching enzyme (SBE) [105]. Of the five enzymes that synthesize starch, GBSS, SBE, DBE, and SSS contribute to amylopectin structure [106]. Zhu et al. [85] also reported that several primary enzymes are involved in the starch synthesis pathway; these enzymes include SUS, UGPase, AGPase, and SS. The biosynthesis of starch differs quantitatively and qualitatively during the development of storage organs [107]. Most biosynthetic enzyme isoforms could be involved in the early development of starch granules, and others become active later [107]. As a percentage of overall starch in the production of storage organs, the amylose content typically rises, suggesting that it is synthesized late compared to amylopectin content. This may be triggered by the pacing of the synthesis of GBSS, since all the SS enzymes are synthesized faster than the GBSS [108].

The commencement of biosynthesis of starch in storage organs involves sucrose mobilization into glucose-6-phosphate and the importation of G6P into the amyloplast by inorganic phosphate (Pi) exchange; by plastidial phosphoglucomutase (Pi) action, glucose-1-phosphate (G1P) is formed by the transformation of G6P [10]. Geigenberger [109] also stated that the formation of ADP-Glc and inorganic pyrophosphate (PPi) occurs through the transformation of Glc-1-P and ATP, catalyzed by ADP-Glc pyrophosphorylase (AGPase), as the first initiated phase. ADP-Glc acts as a glucosyl donor for various classes of SS that extend the α-1,4-connected glucan chains of the two insoluble amylose and amylopectin starch polymers, from which the granule is developed [109]. By transforming glucose 1-phosphate (Glc-1-P) and ATP to ADP-Glc and inorganic pyrophosphate (PPi) in the amyloplasts, AGPase, the first enzyme that triggers the starch biosynthesis pathway, catalyzes the restricting reaction [9]. Regina et al. [10] also reported that the first step of starch synthesis is ADP glucose (ADPG) production, catalyzed by ADPG pyrophosphorylase (AGPase) via ATP activation of G1P. The synthesis of ADP-glucose occurs both in the cytosol and plastids in the developing endosperm of cereal seeds [105]. For starch synthesis, the ADP-glucose produced inside the cytosol is transferred into the plastid and does not have any other metabolic function [105]. The ADP-glucose importation of ADP-glucose into the plastids is achieved by an ADP antiporter (ADP-glucose transporter)/ADP glucose, which is only located in the endosperm of grass [105]. ATP and glucose-1-phosphate synthesize ADP-glucose in a reaction catalyzed by ADPGPPase, releasing pyrophosphate, synthesized amylose, and amylopectin [107]. Inorganic alkaline pyrophosphatase, which is possibly limited to both non-photosynthetic and photosynthetic tissue plastids, eliminates the pyrophosphate formed by ADPGPPase [107]. Riso 16 mutant barley, the small subunit of cytosolic AGPase inactivation (where plastidial AGPase function is not affected), results in decreased aggregation of ADPG and starch in the endosperm with reduction of storage of protein accumulation and seed size occurring at the same time [10]. 3-Phosphoglyceric acid (3-PGA) acts as an enzyme activator, leading to the catalytic activity of the enzymes, and is blocked by inorganic phosphate (Pi) [9]. Oxidation-mediated development of disulfide bridges between neighboring AGPSSs is also restricted to the action of AGPase, which can contribute to reactivation by decreased thioredoxin (or dithiothreitol in vitro) [110]. Hannah et al. [111] observed that in both the tiny *shrunken 2* (*Sh2*) and the huge *brittle 2* (*Bt2*) gene subunits, mutations in AGPase contribute to a significant decrease in starch content. Starch synthase (SS) can be classified as GBSS, which synthesizes amylose and the extra-long chain fraction of amylopectin, and soluble starch synthase (SSS), which synthesizes amylopectin [112]. Geigenberger [109] reported that in plants, five different types of SS groups are known, namely, GBSS, which is responsible for amylose synthesis, and soluble SSs, which range from I to IV and regulate the synthesis of amylopectin. The synthesis of the α-(1,4) linkage between the non-reducing end of the pre-existing glucan chain and the glucosyl moiety of ADP-glucose is catalyzed by SS, triggering ADP release [107]. Both amylose and amylopectin may be used as substrates for SSs in vitro [107]. Amylose is an essentially linear glucan comprising relatively few branches of α-1,6 and generates up to 20–30% of regular starch, whereas amylopectin is extremely branched [109]. Two groups of SBE (SBEI and SBEII), which vary in relation to the extent of the transferred glucan chains and the specificity of the substrate, add branch points [109]. The branching activities of starch branching enzymes, which belong to the α-amylase family, are controlled by Q enzymes by introducing an arrangement that is branched by cleaving the α-1,4-glucan chain in polyglucans and then re-attaching the cleaved chain through an α-1,6-glucan linkage to the acceptor’s chin, thereby forming a branch on the same or another chain [113].

SBE, which hydrolyzes an α-(l,4) linkage within a chain and then catalyzes the creation of an α-(1,6) connection between the decreasing end of the cut glucan chain and another residue of glucose, presumably one from the hydrolyzed chain, creates the α-(1,6) branches in starch polymers [107]. Remarkably, two categories of debranching enzymes are often involved in the synthesis of starch, which split branch points and may have an effect in modifying the branched glucans into a state that can cause crystallization inside the granule [109]. Starch debranching enzymes (DBEs), glucan-modifying enzymes that exist in dual types (PUL and ISA), are also significant in the synthesis of starch [9]. The most significant functional distinction between these ISA types is typically the operation of phytoglycogen and amylopectin by hydrolyzing polyglucan α-1,6 bonds, which play an essential role in altering overly branched chains or eliminating unnecessary amylopectin branches created by branching enzymes to preserve the amylopectin cluster structure [9]. In addition, ISA is likely to have branched amylose chains [9]. PUL naturally cleaves polyglucan α-1,6-linkages in pullulan, while amylopectin has little or no effect on glycogen [114].

**Figure 2:**
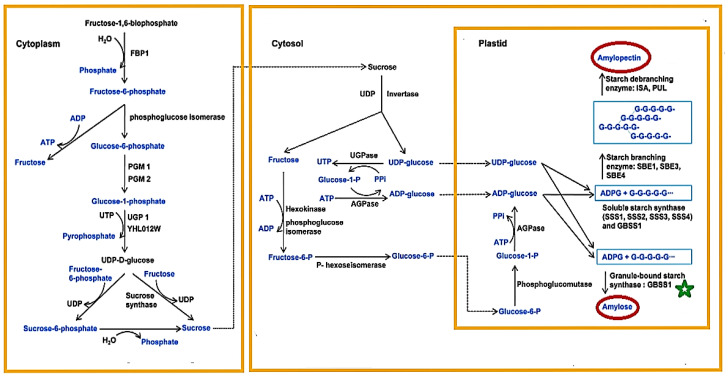
“Main pathway of starch biosynthesis including sucrose synthesis, sucrose degradation and starch synthesis in rice” [8]. “FBP1, fructose-1,6-bisphophatase1; PGM, phosphoglucomutase; AGPase, ADP-glucose pryophosphorylase; PPi, pyrophosphate” [8]. For both gel consistency and amylose content, waxy (GBSSI, marked with a green star) serves as a vital/critical gene, but it has a minor influence on the temperature of gelatinization. The amylose and amylopectin circled in red color are very important in starch modification. Source: Sun et al. [8].

Genetic findings demonstrate that various SS, SBE, and debranching enzyme isoforms are likely to play unique roles in establishing complex starch structures [109]. To synthesize the crystalline matrix of starch granules, they must act in close coordination [109]. Interestingly, SSIII and SSIV are involved in the initiation of starch granules [115]. Recent studies have proposed that the plastidial pathway of starch synthesis occurs in all current green algae and higher plants and that the enzymes involved in starch biosynthesis in higher plants underwent a complicated series of modifications during evolution [116]. The main pathway of starch biosynthesis, including sucrose synthesis, sucrose degradation, and starch synthesis in rice [8], is illustrated in Figure 2.

## 6. Posttranslational Modification (PTM)

PTMs are covalent modifications that alter the principal protein structure in a manner that is sequence-specific, through acylation, phosphorylation, nitration, glycosylation, and ubiquitination, by removal and reversible addition of functional classes [117]. This method is achieved by the induction of a covalent linkage to a new functional group, such as acetyl, phosphate, ubiquitin, or methyl [118]. Proteins are synthesized on ribosomes, generating a nascent polypeptide chain. To generate mature proteoforms that gradually accumulate in plant cells to form the observed proteome, several proteins undergo PTMs [42]. Protein posttranslational modifications greatly enhance functionality, increase proteome diversity, and allow rapid responses, all at reasonably low cell costs [43]. They range from co-translational modifications to position, feature, and signaling enablers and also include stability and degradation markers [42]. PTMs occur on the side chain or on the protein N or C termini and expand the chemical decoration and characteristics of the 20 standard amino acids by changing established functional groups or adding new ones [42]. PTMs play a crucial role in plants via their effects on signaling, enzyme kinetics, protein stability and interaction, and gene expression [43]. After a concise review of the experimental and bioinformatics difficulties of PTM site recognition, position, and occupancy/quantification, a succinct description of the main PTMs and their promising functional effects in plants is presented, with priority on plant metabolism [43]. The center of numerous cellular signaling events is the PTM [119]. Besides the single regulatory PTM, there are other PTMs that act in a coordinated manner [119]. These PTM crosstalks generally function as a modified system for changing cellular responses to the smallest environmental changes [119]. Although PTM crosstalk has been examined in detail in different animals, this aspect is just emerging in plants [119].

Due to the physical–chemical characteristics of reactive amino acids and the cellular atmosphere, including oxygen, pH, metabolites, and reactive oxygen species, or as a result of the activity of particular modifying enzymes, PTMs may occur spontaneously (non-enzymatically) [120]. Significant quantities of proteins and a very sensitive system for their discovery are necessary for identifying PTMs [117]. Kwon et al. [117] also observed that significant quantities of proteins and an extremely sensitive system for their investigation are needed to identify PTMs [117]. Adjacent residues and their exposure to the surface are also important for PTM identification [43]. Over 300 separate forms of PTMs have been reported, and newly discovered types are introduced to the list regularly [121]. Throughout a protein’s life cycle, different PTMs exist [42]. PTMs are subdivided into different groups, which include the addition of peptide groups, amino acid modification, the addition of complex molecules, the addition of chemical groups, and cleavage of proteins via a variety of proteolytic mechanisms [30]. The addition of chemical groups can be grouped into acetylation, redox-based modifications, methylation, and phosphorylation; types of polypeptide addition include SUMOylation, ubiquitination, and other ubiquitin-like protein conjugations; the processes by which complex molecules are added include AMPylation, glycosylation, acylation, prenylation, and ADP-ribosylation; and the direct processes by which amino acids are modified are eliminylation and deamidation [30]. Although protein modification is irreversible, the additions of polypeptides, chemical groups, and complex molecules are sometimes reversible [30]. Glycosylation, acetylation, and phosphorylation are primary types of PTMs, of which phosphorylation is one of the most vital, prevalent, and widely employed protein PTMs used in the course of the research by molecular biologists [122]. Vu et al. [119] also stated that in molecular biology research, the most employed PTM is phosphorylation, and its crosstalk with other forms of PTM has been notably observed, particularly in recent studies. The most critical posttranslational modifications detected in plants include phosphorylation (His, Tyr, Ser, Asp, Thr), S-Nitrosylation (Cys) and nitration (Tyr), acetylation (Lys ε-amine, N-terminal α-amine), deamidation (Asn, Gln), lipidation (S-acetylation, N-myristoylation, prenylation; Gly, Cys, Trp, Lys, N terminal), N-Linked glycosylation (Asp) and O-linked glycosylation (Thr, Lys, Trp, Ser), ubiquitination (N terminal, Lys), sumoylation (Lys), carbonylation (Pro, Thr, Arg, Lys), methylation (Lys, Arg, N terminal), glutathionylation (Cys), oxidation (Cys, Met), peptidase (cleavage peptidyl bond), S-Guanylation (Cys), and formylation (Met) [43] (Table 3).

The reversible phosphate group covalently binds to the hydroxyl group of hydroxyl amino acids such as threonine, serine, and tyrosine but rarely covalently binds to hydroxyproline in protein phosphorylation [123]. Tyr, Thr, and Ser account for PTM phosphorylation with percentages of 1–5%, 15–20%, and 75–80%, respectively [124]. Phosphorylation PTM is the most widely used and most significant in providing proteomic dynamics [125]. PTM, particularly phosphorylation of protein, has long been regarded as a crucial regulatory mechanism that regulates transcription factors [126,127]. Its discovery in recent times has propelled decades of potential phosphoproteome studies [30]. Protein phosphorylation has arisen as one of the main PTMs. It plays a key role in DNA replication, cell signaling and differentiation, gene expression, and enzyme activity increases or decreases; it also changes other biological effects, controlling cell proliferation and enlargement, phytohormone biosynthesis and signaling, plant disease resistance, grain-filling, and development of rice seed quality [128].

C3 phosphorylation accounts for 20 to 30% of phosphate monoesters, while about 70 to 80% of phosphate monoesters are attached to the C6 position of the glucosyl unit [129]. In order to catalyze the phosphorylation of starch, two forms of glucan water dikinases have been employed: glucan water dikinase (GWD1) and phosphoglucan water dikinase/glucan water dikinase 3 (PWD/GWD3) [14]. Specifically, GWD1 phosphorylates starch at the C6 position, catalyzing the transition of β-phosphate from ATP to a residue of glucosyl, and then PWD identifies the pre-phosphorylated glucan C6 and ultimately phosphorylates hydroxyl C3 [130]. Glucan water dikinase (GWD1) and phosphoglucan water dikinase/glucan water dikinase 3 (PWD/GWD3) catalyze starch phosphorylation, which occurs naturally during starch metabolism in plants [14]. The only identified naturally occurring PTM of starch is phosphorylation [131]. Phosphorylation often liaises with other forms of PTMs in the cell, most often for protein turnover and protein function attenuation [33]. Nabeshima et al. [132] reported that the incorporation and quantity of phosphate groups differ pertaining to the type of starch, based on the source of the starch, amylose–amylopectin ratio, purity of the starch, microstructural features, conditions during phosphorylation, and various phosphate salts. GWD1 phosphorylation disrupts the starch surface crystalline structure and enhances the hydrolytic action of plastidial β-amylases [133].

The degree of phosphorylation of starch differs greatly because of the sources of starch in the plant [134]. Hebelstrup et al. [131] reported that phosphate levels are higher in tuber starch than in cereal starch. Compared with leaf starch, the content of phosphate detected in starch deposited in seeds is very low [135]. Thorough study on modification of *Wx* has rendered it easier to identify more suitable target locations for editing and enabled the amylose content of crops to be controlled more specifically in a more effective manner [62]. The reduction of phosphorylation at the Ser-34 position affects the activity of GBSSI, synthesizes amylase, and leads to amylose content reduction [136,137]. Phosphate ester removal is important for starch degradation [135]. Liu et al. [50] reported that the substitution of Asp165/Gly165 had no noticeable influence on the activity of GBSSI in vitro; nevertheless, it remarkably caused a reduction in GBSSI binding to the starch granules, which led to amylose content reduction in rice. More knowledge of GBSS posttranslational and transcriptional control may lead to the discovery of new mechanisms that affect amylose content [138]. LC-MS/MS was used to identify the phosphorylation sites in GBSSI [137]. Non-amylolytic GWD glucan phosphorylation is necessary for the turnover of plant starch [135]. Huang et al. [62] reported that in the waxy locus, other sites might be targets for PTM modification or translation (e.g., the stability of protein, starch binding capacity, phosphorylation, ADP-glucose binding, and codons that control the enzymatic function of GBSSI). Zhang et al. [24] located nine phosphorylated PTM sites in rice GBSSI, but only Ser415P had an altered level of phosphorylation, leading to moderate amylose content. One approach for enhancing bakery product quality is through the phosphorylation of rice [139]. Kringel et al. [139] reported that wild-type rice had a lower pasting temperature and higher breakdown rate than phosphorylated rice. Phosphorylation results in a substantial decrease in the paste synthesis and retrogradation of rice flour [139]. Phosphorylated rice flour used for bread baking demonstrated a hardness reduction at all the tested storage temperatures and had an impact on the bulkiness, texture, and color of rice bread, indicating the feasibility of utilizing phosphorylated rice flour in gluten-free bread [139]. Gluten’s viscoelastic properties cannot be improved without modifying the fraction of protein, as shown by the use of gluten-free cereals in dough systems [140]. In proteomic studies of gluten proteins, PTM is commonly used to explore the modification of protein [141]. The linearity of amylopectin and amylose molecules can be interrupted by the use of negatively charged phosphate classes [139]. The second cys (Cys529) in rice GBSSI is conserved in plant GBSSIs, and insertions of sequence were likewise found around this cysteine [142]. In the *Poaceae* family, the first cys (Cys337) in GBSSI of rice, rye, corn, sorghum, wheat, and barley is conserved, whereas in non-Poaceae plants such as soybean, pea, *Arabidopsis thaliana*, cassava, kidney stone, sweet potato, and potato, it is substituted with valine [142]. In cultivated rice, the combination of ‘GC’ (leucine) at SNP4 and ‘G’ (valine) at SNP3 is necessary for the development of L-type rice starch, which has a higher GT than S-type rice starch [143]. The conversion of L-type starch to S-type starch is caused by changing leucine to phenylalanine or valine to methionine, which leads to low GT [143]. Consumers prefer low-GT rice because of its cooking quality [143]. It is worth noting that in poaceous GBSSIs, disulfide bonds are detected, including in most cereal plants that store large amounts of starch in their seeds [142]. The disulfide bridges cause restricted domain movement, which could help to improve the efficiency of starch biosynthesis [142]. Liu et al. [50] reported that via a posttranslational approach, *Wx^hp^* directly affects the *Wx* gene and subsequently influences Haopi amylose content. In addition, by editing the CpG site in the waxy promoter, DNA methylation modification can give rise to rice with preferable eating quality and healthy rice with minimized GI [62]. DNA methylation is linked to amylose and amylopectin synthesis, and altered methylation can affect gene expression [144]. Lysine acetylation has been observed in many primary enzymes in the starch synthesis pathway, including GBSSI, AGPS, SBE3, AGPLar, SBE1, and Pho1 [38]. Amylose chains can depolymerize as a result of acetylation [145]. Out of 247 proteins, 421 malonylated lysine locations were observed and perform a crucial role in multiple essential metabolic processes, including lipid metabolism, central carbon metabolism, photosynthesis, and biosynthesis of starch [146]. Lysine, arginine, and charged amino acids in the lysine flanking position are favored residues [146]. In recent years, lysine succinylation has been recognized as a posttranslational modification (PTM) [147]. Because of the bulkier structural modifications and more important charge variations on the changed lysine residue, succinylation may have a greater practical effect than acetylation [147]. During the development of grains, cereals undergo various PTMs of proteins [148]. Many of the class of enzymes involved in biosynthesis of starch are regulated and coordinated and in a range of ways from gene expression to different posttranslational mechanisms including protein phosphorylation and redox modulation [149]. Meng et al. [147] also observed that the enzymes involved in starch biosynthesis and regulatory pathways, including starch branching enzymes (OsBEI and OsBEIIb), ADP-glucose pyrophosphorylases (OsAGPS2 and OsAGPL2), sucrose synthase (SUS2 and SUS3), starch debranching enzymes (OsPUL), UDP-glucose pyrophosphorylase (UGP), phosphoglycerate mutase (PGM), and starch phosphorylase (OsPHOL), in developing rice seeds undergo succinylation. Surprisingly, heavy succinylation was found on major seed storage proteins, as well as key enzymes involved in central carbon metabolism and starch biosynthetic pathways for the development of rice seed [147]. In order to improve starch quality, most of the starch biosynthesis proteins listed above can be subjected to crotonylation, acetylation, 2-hydroxyisobutyrylation, and malonylation [147]. Multiple (PTM-dependent) approaches carefully control all steps of CO_2_ fixation and starch metabolism by balancing the rate of biosynthesis of starch with the availability of carbon and energy in numerous tissues of plants under different environmental conditions [150].

In wheat, Chen et al. [137] detected three GBSSI phosphorylation sites, namely Thr323, Ser34, and Ser450, and three phosphorylation sites in SSIIa, namely Ser776, Tyr358, and Ser228. In both SN119 and ND5181, five starch synthesis enzymes, including SSI, GBSSI, SBEI, SBEII-a, and SSII-a, were phosphorylated, and their phosphorylation sites were conserved [46]. In particular, SSIIa and GBSSI had eight and nine phosphorylated sites, respectively, showing that in starch biosynthesis, many conserved phosphorylated sites play essential roles [46]. In addition, the starch granule-binding proteins were heavily stained with Pro-Q Diamond at five developmental levels [46]. At all five phases, SSI, GBSSI, SBEIIa, and SSIIa were phosphorylated, with phosphorylation levels in ND5181 and SN119 being akin [46]. The characterization of starch granule-binding proteins (SGBPs) by phosphoproteome under water deficit treatment showed reduced phosphorylation levels of major starch synthesis enzymes, notably for GBSSI, SSIII, and SSII-a, that led to total starch reduction [137]. The enzymes involved in starch phosphorylation are attractive candidates for the regulation of flux via degradation of starch [151]. Specifically, SSIII-a site Ser837, GBSSI protein site Ser34, and SSII-a site Tyr358 demonstrated a slight decrease in phosphorylation under water-deficit treatment compared to under well-watered treatment [137]. A higher rate of phosphorylation of starches is characterized by a higher turnover of phosphate groups that exhibit transient esterification [152]. Only in B-type starch granules does the SSI-1 phosphorylation occur [153]. SSI-1 predominantly synthesizes the smallest glucan chain with a DP of approximately 10 glucosylic units or less [154]. The level of phosphorylation at the 36 phosphorylated sites of 19 phosphoproteins was substantially altered [46]. Twenty-two and fourteen phosphorylated sites were discovered in SN119 and ND5181, respectively, out of the thirty-six phosphorylated sites [46]. Glucose-6-phosphate isomerase ser600 was only observed to be phosphorylated in ND5181, while the vacuolar cation or proton exchanger sites Tyr243 and Tyr249 were phosphorylated in SN119 only [46]. Chen et al. [46] observed that the absence of amylose did not influence SGBP expression and the phosphorylation of SGBP is involved in amylopectin biosynthesis.

In maize, a phosphorylation-specific dye study of the granule phosphoproteome showed that starch phosphorylase, GBSS, and BEIIb are phosphorylated as they exist in the granule [91]. Crosslinked maize starch procured by different phosphorylation approaches displayed an elevated percentage reduction in the peak viscosity stretch from 97.1 to 99.6% relative to pulse starches, suggesting that the impact of crosslinks on maize starch pasting properties was more noticeable than that on pulse starches [155]. The probability that starch metabolic enzymes present in granules is controlled by protein–protein interactions and/or modifications was observed [91]. In maize and wheat amyloplasts, the soluble types of starch phosphorylase and BEIIb accept the transition by marking the ATP of a radioactive phosphate group [91]. The crosslinked starch with STMP aqueous solution had a less noticeable effect on the improved crystalline stability than STMP semidry and POCl_3_ aqueous solution [155]. STMP-aqueous only increased Tc in pulse starches and substantially increased Tp, To, and Tc in maize starch, indicating that crosslinking systems and crosslinking agents have different impacts on different types of starches [155]. Phosphorylation crosslinking considerably improves the pasting temperature, breakdown reduction, gelatinization temperature increase, and thermal stability in various forms, which may permit broader applications in corn, field peas, and faba beans [155].

In Arabidopsis, the low MNF-Pin-39 variety activity indicates that the replacement of Ser-570Tyr is counterproductive to activity and, considering significant GBSS abundance, is attuned with the very low amylose content [156]. The substitution of the amino acid in the GBSS variant does not seem to be harmful to the operation but rather inhibits in vivo amylose synthesis through other techniques; Gn2–3 and TueSB30-3 bear the same GBSS allele, which encodes the substitution of two amino acids (Asn-430Ser and Gly-394Glu) [156]. The lack of amylose means that one or both amino acids are required for in vivo GBSS action [156].

In potato tubers, starch phosphorylation can induce both starch degradation and starch synthesis [157]. Due to the existence of phosphate groups, wild-type potato starch produces a transparent paste, while an increased level of phosphorylation steadily reduces the clarity of the paste [158]. One out of 200–300 glucose units of amylopectin in potato starch is phosphorylated [159]. Phosphate groups may be bound to a glucose residue at the C3 or C6 positions [159]. Potato plants with decreased amounts of R1 protein exhibit much less starch phosphorylation, suggesting that this protein is essential for starch phosphorylation [134]. Phosphorylation carried out at a low substitution degree leads to a significant increase in the swelling power and solubility, whereas the swelling power and solubility decrease steadily with increasing substitution levels [158]. As the degree of phosphorylation increases, viscosity values decrease, while at the lowest degree of substitution of various starch forms apart from maize amylose, the viscosity increase [158]. Therefore, the preparation of phosphorylated starches with very low levels of replacement is suggested to be the best property for paste transparency [158]. Potato tuber reserve starch is another example of a starch phosphorylation feature during starch synthesis [135]. Minor changes in the metabolism of storage starch were recorded in transgenic potato lines with downregulation of *StGWD* [160]. This line of antisense leads to the yield of more, but smaller tubers, and both the viscosity of the starch and the quality of amylose are influenced [135]. In the endosperm amyloplast in barley, *StGWD* overexpression heterologously leads to a 10-fold increase in grain starch-bound phosphate [161]. The development of transformants in which the native catalytic subunit of AGPase (AGPB) is substituted by a modified type of AGPB in which Cys-82 is modified to prevent the formation of an intermolecular bridge is needed for the final genetic proof of posttranslational redox regulation, which is responsible for the inhibition of starch synthesis after tuber detachment [162].

Nabeshima et al. [132] observed the phosphorylation of sago-starch at pH 9.5 and pH 9 at different concentrations of sodium trimetaphosphate (2% and 5%), and they reported that the phosphorylation at pH 9.5, displayed higher cold-paste viscosity and lower hot-paste viscosity than the phosphorylated sago-starches at pH 9 with sodium trypolyphosphate. STMP-semidry reduced the onset gelatinization temperature but greatly elevated the peak and conclusion gelatinization temperatures in all starches [155]. With a rise in the number of phosphate groups introduced in the course of phosphorylation, the values for adhesiveness and firmness declined [132]. The transcriptional expression modifications of many genes that synthesize amylopectin, such as *SBEII-b*, *SSIII*, and *AGPL I*, were substantially decreased at 15 DPA, which is in line with GBSSI, indicating that the impact of drought stress on the biosynthesis of starch occurs at the stage of transcription, translation, and phosphorylation of proteins. Starch crosslinking greatly increases the temperature of gelatinization, whereas the enthalpy is not significantly affected [132]. The To, Tp, and Tc changes in field pear, faba bean, and regular corn showed that field pear had the highest To, Tp, and Tc, followed by faba bean and regular corn [155]. Owing to the restricted enlargement and decreased hydration of starch granules in rice, retrogradation and gelatinization are delayed [132].

## 7. Conclusions

Significant advancements have been made through the downregulation and overexpression of genes, which are comparatively brute force methods that sometimes contribute to unwanted trade-offs between plant development and stress resilience, and a better way of improving crops may be achieved through the posttranslational modification of sites or regulatory enzymes that regulate them because of their significance. The waxy locus controls both the non-waxy and waxy rice phenotypes. Active granule-bound starch synthase is essential for amylose synthesis, while amylopectin is synthesized as a result of a complex pathway involving various isoforms of starch-debranching enzymes, starch branching enzymes, and starch synthase. An interaction study of 18 genes involved in gelatinization temperature, starch synthesis, gel consistency, and amylose content revealed that waxy and *ALK* are the major genes responsible for determining the nature of rice cooking and eating quality by influencing the gel consistency, amylose content, and temperature of gelatinization. Rice starch can be altered into various forms by either reducing or increasing the amylose content, depending on consumer preference and region. The majority of consumers prefer waxy and low-amylose rice varieties because of their cooking and eating qualities. It is regionally desired and selected for consumption as cooked rice because of its softness and stickiness. To meet the market demand, which is increasing every day, it is important to focus more on yield improvement of *Wx* and low-amylose rice because increases in rice production rely largely on the improvement of yield, which could be achieved by posttranslational modification rather than an extension of the planting field. Therefore, there is a need for government, private, and parastatal organizations to invest more in research to improve the palatability of rice for consumers.

## Figures and Tables

**Table 1:** Genes involved in starch modification.

Genes	Function	References
*Wx*, *AGPlar*, *PUL*, *SSI*, *ALK* (*SSII-3*)*, SSIII-2*	Amylose synthesis	[69]
*Wx*, *AGPiso*, *SBE3*, *ISA*, *ALK* (*SSII-3*)	Gel consistency	[69]
*ALK* (*SSII-3*), *Wx*, *SBE3*, *ISA*, *SSIV-2*	Gel temperature	[69]
*SBE1*, *SBE3*	Grain palatability	[8]
*SBE1*, *SBE3*, *SBE4*, *ISA*, *PUL*	Amylopectin synthesis	[92]
*BEI*, *BEIIb*, *BEIIa*, *SSIIa*, *SSIIIa*, *SSI*, *PUL*, *ISA1*	Amylopectin synthesis	[93,94]

**Table 2:** Internal starch granule associated proteins in crops [91].

Crops	Enzymes Identified in Starch Granules	References
Rice	BEIIb (82 kDa), SSIIa (86 kDa), SSI (72 kDa), GBSS (60 kDa)	[95]
Wheat	BEIIa, BEIc (SGP-145, 145 kDa), BEIc (SGP-140, 140 kDa), BEIIb (SGP-2, 92 kDa), SSIIa (SGP-A1, 115 kDa), SSIIa (SGP-D1, 108 kDa), SSIIa (SGP-B1, 100 kDa), SSI (SGP-3, 80 kDa), GBSS (60 kDa)	[96,97]
Barley	BEIc (140 kDa), BEIIb (93 kDa), SSIIa (87 kDa), SSI (71 kDa), GBSS (60 kDa)	[98,99]
Maize	BEIIb (85 kDa), SSIIa (86 kDa), SSI (76 kDa), GBSS (60 kDa)	[100,101]
Potato	R1 (160 kDa), SSII (92 kDa), GBSS (60 kDa)	[102,103]
Pea	R1 (160 kDa), BEI (114 kDa), BEII (100 kDa), SSII (77 kDa), GBSS (60 kDa)	[103,104]

**Table 3:** The most critical posttranslational modifications detected in plants [43].

Type of PTM (Reversible If Asterisk)	Enzymatic or Spontaneous (Nonenzymatic)	Comment on Subcellular Location and Frequency
Phosphorylation (His, Tyr, Ser, Asp, Thr)	Enzymatic	Phosphorylation of His and Asp have low frequency
S-Nitrosylation (Cys) and nitration* (Tyr)	Spontaneous (RNS), but reversal by thioredoxins is enzymatic for Cys	Throughout the cell
Acetylation (Lys ε-amine, N-terminal α-amine)	Enzymatic	In mitochondria, very little N-terminal acetylation, but high Lys acetylation; Lys acetylation correlates to [acetyl-CoA]
Deamidation (Asn, Gln)	Spontaneous, but isoAsp reversal is enzymatic by isoAsp methyltransferase	Throughout the cell
Lipidation (S-acetylation,N-myristoylation*, prenylation*; Gly, Cys, Trp, Lys, N terminal)	Enzymatic	Not (or not often) within plastids, peroxisomes, mitochondria
N-Linked glycosylation (Asp); O-linked glycosylation (Thr, Lys, Trp, Ser)	Enzymatic	Only proteins passing through the secretory system; O-linked glycosylation in the cell wall
Ubiquitination (N terminal, Lys)	Enzymatic	Not within plastids, peroxisomes, mitochondria
Sumoylation (Lys)	Enzymatic	Not within plastids, peroxisomes, mitochondria
Carbonylation* (Pro, Thr, Arg, Lys)	Spontaneous (ROS)	High amounts in chloroplast and mitochondria
Methylation (Lys, Arg, N terminal)	Enzymatic	Chloroplasts and histones (nucleus); still not fully explored
Glutathionylation (Cys)	Enzymatic	High amounts in chloroplasts
Oxidation (Cys, Met)	Spontaneous (ROS) and enzymatic (by PCOs), but reversal is enzymatic by thioredoxins, Met sulfoxide reductases, and glutaredoxins, except if double oxidized	High amounts in chloroplast and mitochondria
Peptidase* (cleavage peptidyl bond)	Enzymatic	Throughout the cell
S-Guanylation (Cys)	Spontaneous (RNS)	Rare; 8-nitro-cGMP is signaling molecule in guard cells
Formylation (Met)	Spontaneous, but deformylation by peptide deformylase is enzymatic	All chloroplasts and mitochondria-encoded proteins are synthesized with initiating formylated Met

## Data Availability

Not applicable.
